# 450. Antibiotic Fills and Readmissions After Patient-Directed Discharge in Drug Use-Associated Endocarditis: A National Medicaid Study

**DOI:** 10.1093/ofid/ofaf695.149

**Published:** 2026-01-11

**Authors:** Fiona Elizabeth Gispen, Shashi Kapadia, Kenneth Karan, Yuhua Bao, Ximena A Levander, Elaine Werthington, Todd Korthuis, Benjamin Eckhardt

**Affiliations:** Weill Cornell Medicine, Brooklyn, NY; Weill Cornell Medical Center, New York, New York; Weill Cornell Medicine, Brooklyn, NY; Weill Cornell Medicine, Brooklyn, NY; Oregon Health & Science University, Portland, Oregon; Cornell Universtiy, Ithaca, New York; Oregon Health and Science University, Portland, Oregon; NYU Langone Health, New York, New York

## Abstract

**Background:**

Antibiotic strategies for drug use–associated infective endocarditis (DUA-IE) continue to evolve, but limited real-world data exist regarding treatment after patient-directed discharge (PDD). This study examines antibiotic use patterns, clinical characteristics, and readmissions among DUA-IE patients with PDD as compared to conventional discharges.

**Figure:**
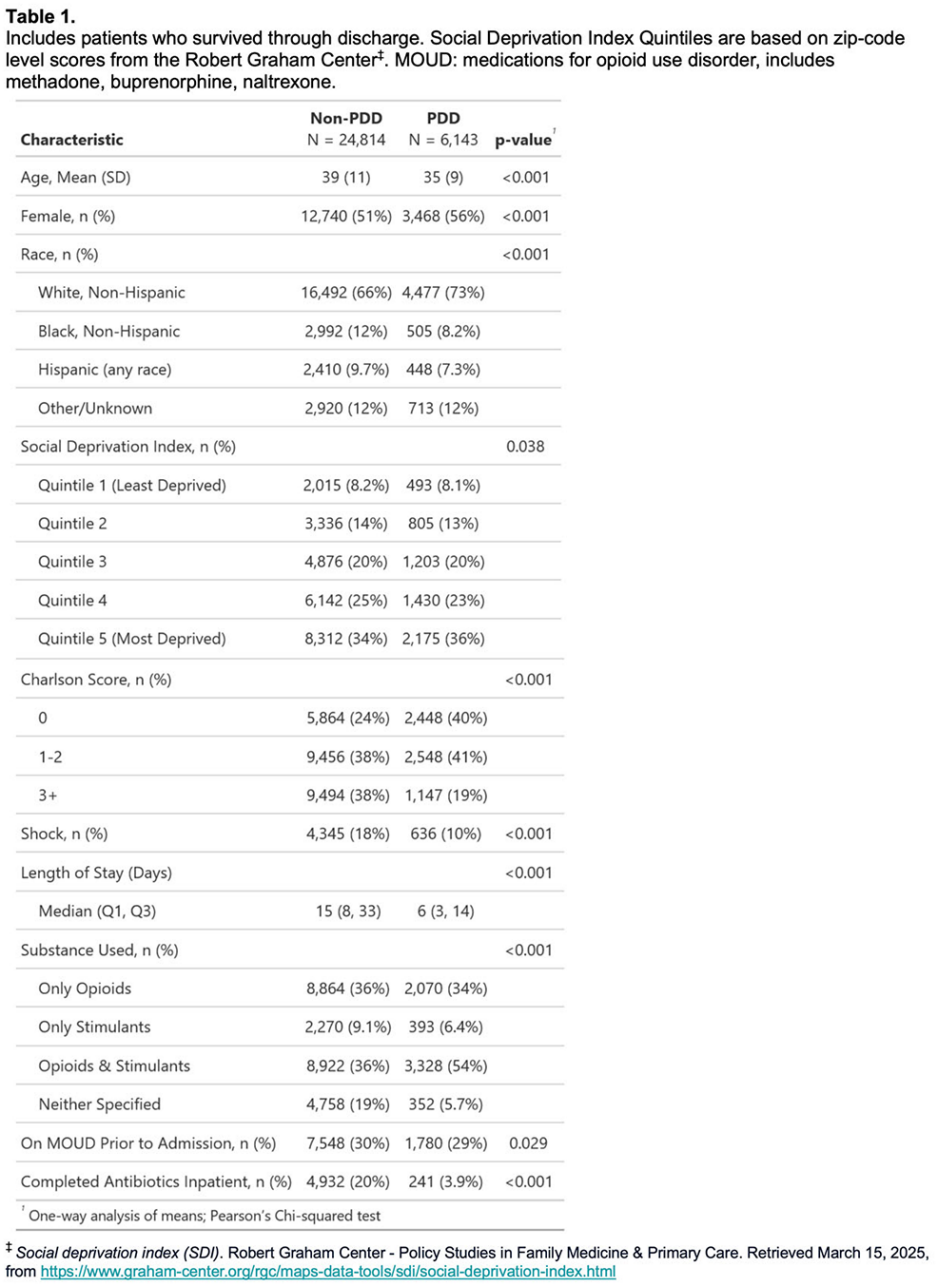

**Figure:**
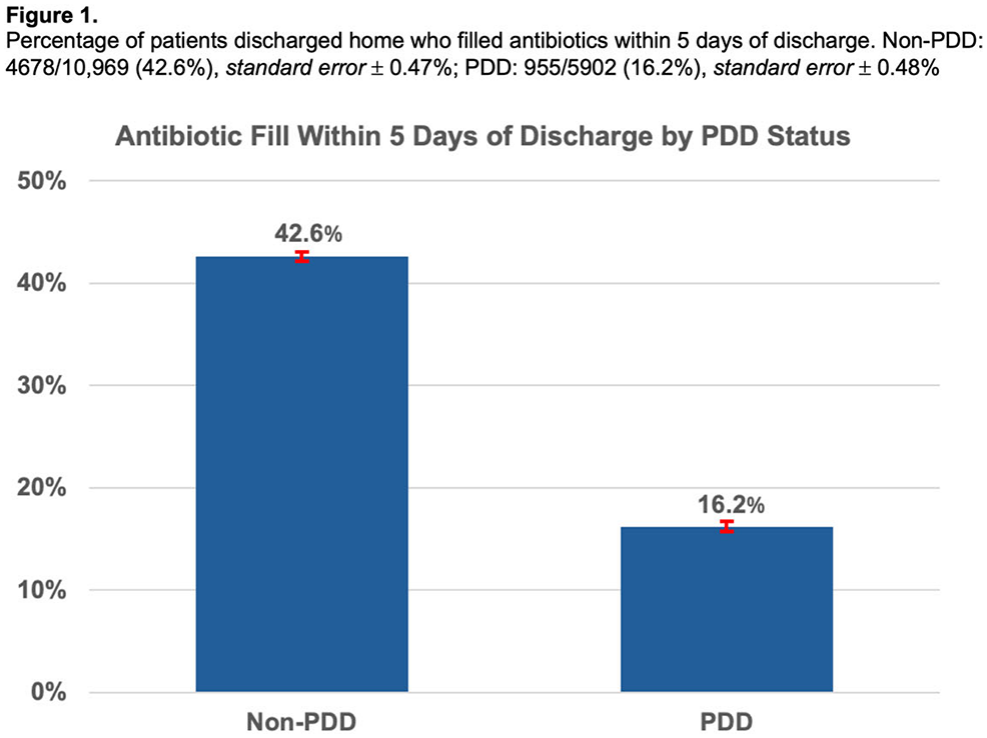

**Methods:**

Using national Medicaid data (July 2016–June 2021), we identified adults aged 18–64 hospitalized with new episodes of DUA-IE. We identified endocarditis and drug use using ICD-10 codes, and we required continuous Medicaid enrollment for 6 months before through 1 month after hospitalization. We compared demographic and clinical characteristics, post-discharge antibiotic fills, and 30-day readmissions by PDD status. For those discharged to non-facility settings before completing recommended therapy (4-6 weeks based on pathogen), we estimated post-discharge antibiotic fill rates.

**Figure:**
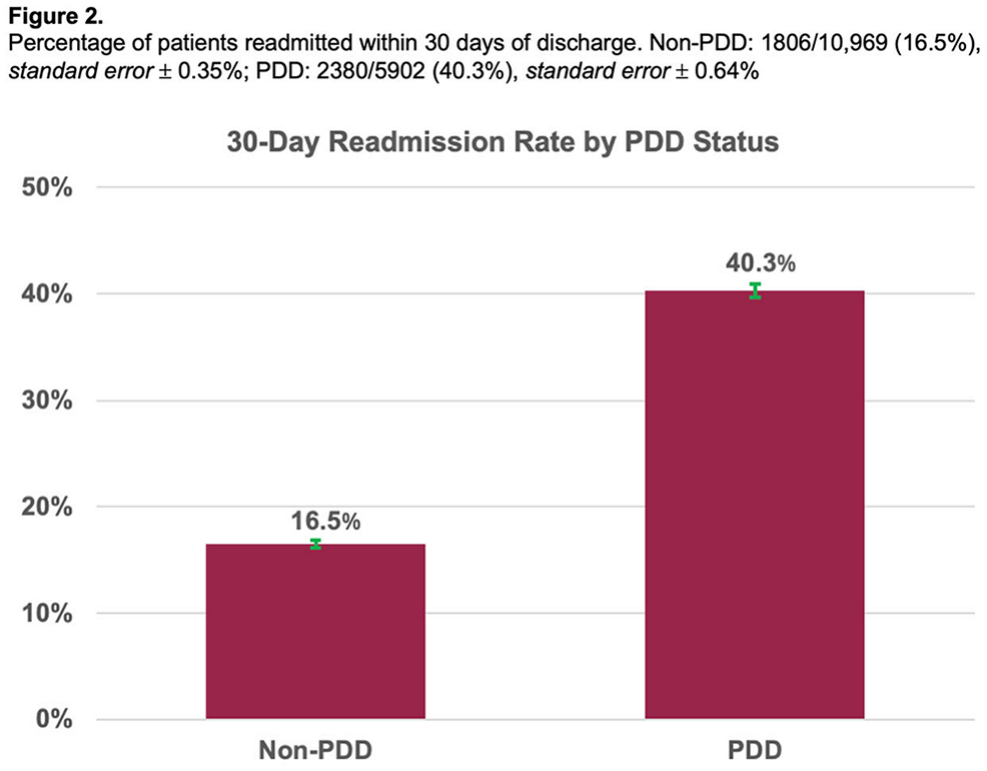

**Figure:**
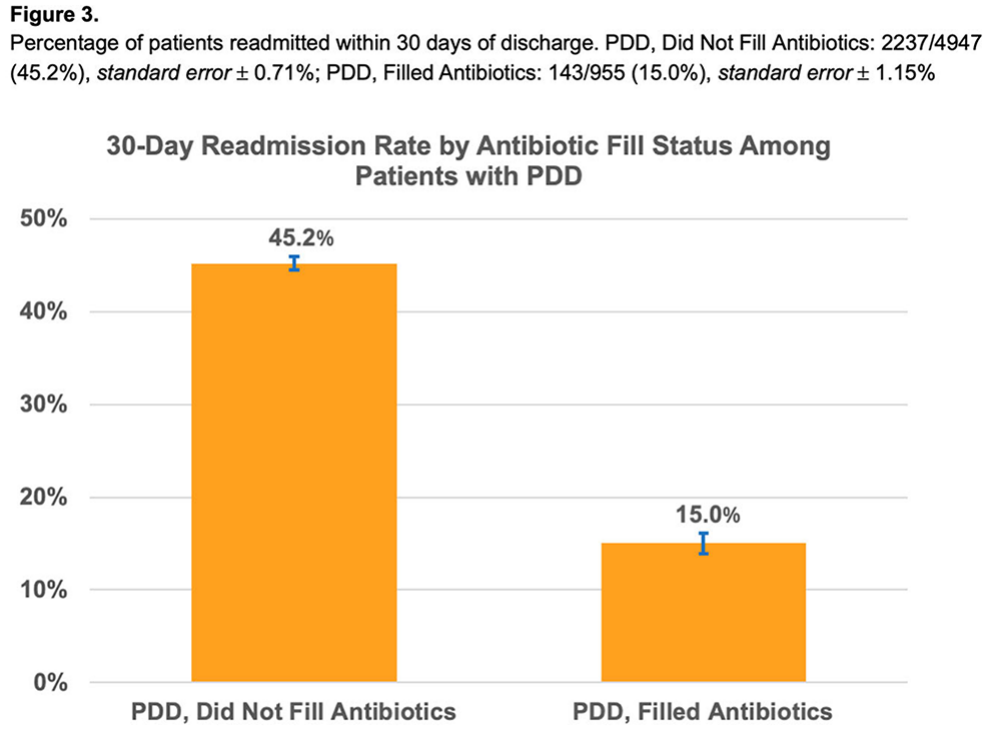

**Results:**

Among 33,298 DUA-IE episodes, 30,957 (93%) survived to discharge. Of these, 5,173 (16.7%) completed treatment while inpatient. Among the 25,784 discharged before completing treatment, 5,902 (22.9%) left via PDD. PDD patients were younger, more often female, less sick, and had more dual opioid/stimulant use (Table 1). Among those discharged to non-facility settings before completing treatment, 16.2% of PDD patients filled antibiotics within 5 days, compared to 42.6% of non-PDD patients (p< 0.001), and 40.3% were readmitted within 30 days, compared to 16.5% of non-PDD patients (p< 0.001) (Figures 1 and 2). Among PDD patients, 45.2% of those who did not fill antibiotics were readmitted, compared to 15.0% of those who did (p< 0.001) (Figure 3).

**Conclusion:**

In this national cohort of Medicaid enrollees with DUA-IE, patients with PDD had lower post-discharge antibiotic fill rates and higher 30-day readmission rates than those without. Among PDD patients, filling antibiotics was associated with reduced readmissions. These findings highlight the need for enhanced strategies to support treatment continuity and improve outcomes in this vulnerable population – including planning for potential PDD, bedside medication delivery, and expanded use of long-acting antibiotics.

**Disclosures:**

All Authors: No reported disclosures

